# Prevalence and Associated Factors of Pathological Internet Use and Online Risky Behaviors Among Japanese Elementary School Children

**DOI:** 10.2188/jea.JE20200214

**Published:** 2021-10-05

**Authors:** Masaaki Yamada, Michikazu Sekine, Takashi Tatsuse, Yukiko Asaka

**Affiliations:** Department of Epidemiology and Health Policy, School of Medicine, University of Toyama, Toyama, Japan

**Keywords:** disharmony, family, gaming disorder, Internet addiction, problematic

## Abstract

**Background:**

Little is known about pathological Internet use (PIU) and online risky behaviors among elementary school children. We aimed to investigate the prevalence and factors associated with PIU and online risky behaviors.

**Methods:**

A school-based cross-sectional study was conducted in Toyama, Japan in 2018. The study included 13,413 children in the 4th–6th grades (mean, 10.5 years old) from 110 elementary schools (61.1% of elementary schools in Toyama). We assessed PIU using Young’s Diagnostic Questionnaire (YDQ) and risky behaviors. Poisson regression analysis was conducted.

**Results:**

Totally, 13,092 children returned questionnaires (response rate 97.6%). The prevalence of PIU was 4.2% and that of each risky behavior was as follows: 21.6% for spending money online, 6.6% for uploading personal movies, 5.2% for interpersonal issues, and 2.4% for having met strangers. PIU was significantly associated with boys (prevalence ratio [PR] 1.26; 95% confidence interval [CI], 1.04–1.52), skipping breakfast (PR 1.43; 95% CI, 1.14–1.79), Internet time (for 2∼3 h, PR 3.49; 95% CI, 2.63–4.65; for 3∼4 h, PR 4.45; 95% CI, 3.27–6.06; and for ≥4 h, PR 8.25; 95% CI, 6.45–10.55), physical inactivity (PR 2.63; 95% CI, 2.00–3.47), late bedtime (PR 1.86; 95% CI, 1.45–2.39 for ≥11 p.m.), no rules at home (PR 1.22; 95% CI, 1.01–1.46), no child-parent interaction (PR 1.37; 95% CI, 1.06–1.77), and no close friends in real life (PR 1.69; 95% CI, 1.30–2.19).

**Conclusions:**

PIU and risky behaviors were not rare among the elementary school children. Besides unhealthy lifestyles, social and family environments were associated with PIU. Having child-parent interaction and helping children develop close friendships in real life are effective deterrents to PIU.

## INTRODUCTION

The growth of the Internet has dramatically changed human lives, and the number of Internet users has soared in Japan. Japanese government data show that in 2018, the penetration rate of Internet use was more than 95% among people aged 13–49 in 2018 and approximately 70% among children aged 6–12.^1^ The Internet has become an essential part of daily life for children as well as adults. It is a useful tool that can provide indefinite information and let people interact conveniently with their family, friends, classmates, or work mates. However, excessive or inappropriate Internet use can cause pathological Internet use (PIU, also known as “Internet addiction” in general), which is an impulse-control disorder that does not involve intoxicants and results in moderate to severe problems in daily life^2^ or can lead to risky situations, such as interpersonal issues.

Nationwide surveys on PIU among junior and high school students have been repeatedly conducted in Japan. A survey using Young’s Diagnostic Questionnaire (YDQ)^2^ demonstrated that the prevalence of PIU was 10.6% in junior high school boys and 14.3% in girls in 2017.^3^ These rates were nearly doubled from the previous survey conducted in 2013. PIU among adolescents is becoming a social concern. Other national reports about online risky behaviors or troubles, such as interpersonal issues (arguing, fighting, cyber harassing, or cyberstalking), showed that 14.0% of elementary students, 33.8% of junior high students, and 56.0% of high school students had experienced at least one form of such troubles.^4^ Although most studies on PIU and risky behaviors have predominantly targeted junior high or high school-aged adolescents,^5^^,^^6^ little is known about such Internet-related problems among elementary school children. The peak prevalence of PIU in children was reported to occur between age 14 and 15 in Japan.^7^^,^^8^ As Internet penetration is high even among elementary school children,^1^ a study targeting them should be accumulated for preventive education at an early stage of life.

Previous studies conducted in adolescents have reported that PIU was associated with unhealthy lifestyles including sleep deprivation and skipping breakfast,^8^^–^^10^ psychological states such as aggressive behavior and depression, and social factors such as familial disharmony and problems with peers.^5^^,^^6^ There have been two previous studies assessing PIU in elementary school.^7^^,^^11^ Li et al demonstrated the descriptive data of PIU prevalence (6.3%) from the nationwide representative study in China.^11^ Meanwhile, Takahashi et al reported that from 3,845 children, those with PIU (including maladaptive Internet use) exhibited more severe depression and decreased health-related quality of life than those with adaptive Internet use.^7^ However, information about lifestyle, family, and social factors were lacking in these studies. We hypothesized that clarifying the comprehensive association of PIU with lifestyle and family and social factors could give us valuable information for the early detection and prevention of PIU. Therefore, we aimed to (1) clarify the prevalence of PIU and online risky behaviors, and (2) explore the association between PIU, children’s lifestyle, and their family and social environments in a large-scale study targeting elementary school children.

## METHODS

### Participants and the Toyama Safe Internet Use Survey

We conducted a school-based cross-sectional study among children who participated in the Toyama Safe Internet Use Survey in 2018. Toyama Prefecture is located approximately in the center of Honshu, Hokuriku District, and has a population of approximately 1 million. Called by the Toyama Prefecture Education Board, 110 elementary schools out of 185 in Toyama Prefecture (as of 2018, 61.1% of elementary schools in Toyama) agreed to join the survey and in total 13,413 children participated. Nowadays, almost all elementary schools have a major concern about Internet use among children, and each school is conducting a survey or setting an abstinence day from internet use, called “No media day.” The rest of the schools (38.9%) had already conducted a similar survey on Internet use, so they did not join when they were contacted by the Education Board. The survey targeted children in the 4th–6th grades because they were required to reflect on their current Internet use and think about how to use the Internet in moderation in the future. An anonymous, self-reported questionnaire was distributed to all children via every school to examine their lifestyle, Internet use, and health. Children filled them out in the classroom and returned. The contents and overall purpose of this survey were explained by classroom teachers and informed consent (assent) was obtained from children and their parents. Participation was voluntary, and the parents or guardians were provided the opportunity to opt out of participation. The study protocol was approved by the institutional ethical committee at University of Toyama.

### Measures

Our questionnaires consisted of basic characteristics, lifestyle, Internet use, health, and family and social factors. Information on lifestyle included breakfast, physical activity, and sleep habits. Responses to breakfast were coded into two groups as follows: “having breakfast every day” or “not having breakfast every day,” the latter of which was defined as “skipping breakfast.” Physical activity was divided into three levels: “very often,” “often,” or “rarely to almost never.” Response to wakeup time was categorized into three groups: “<6:30 a.m.,” “6:30 a.m. to <7:00 a.m.,” or “≥7:00 a.m.”; response to bedtime was divided into three: “<10:00 p.m.,” “10:00 p.m. to <11 p.m.,” or “≥11 p.m.” The validity of the lifestyle questionnaire that assessed physical activity and sleep habits was examined in our previous study.^12^^,^^13^

We asked for the total time of Internet use at home on a weekday, the severity of PIU, and Internet-related risky behaviors. Answers on Internet time were categorized from 1 to 6, as follows: 1, no or almost no Internet time; 2, <1 hour; 3, 1 hour to <2 hours; 4, 2 hours to <3 hours; 5, 3 hours to <4 hours; and 6, ≥4 hours. Thereafter, we collapsed these categories into four: <2 hours, 2 to <3 hours, 3 to <4 hours, and ≥4 hours. To assess PIU, we used a Japanese version of the Diagnostic Questionnaire consisting of eight items, with “yes” or “no” as the answers, and scores ranging from 0 to 8.^2^ A higher score indicates a more serious grade of PIU. Following previous studies,^2^^,^^14^ we categorized each child into three groups based on the YDQ total score as follows: PIU (score ≥5), maladaptive Internet use (score 3 to 4), and adaptive Internet use (0 to 2). Regarding online risky behaviors, we asked about children’s experience of spending money for playing online games or purchasing items, causing interpersonal issues, uploading personal movies, and having met strangers encountered online in real life. Overspending on online games and interpersonal issues were cited as common Internet-related problems in the national survey among junior high and high school students.^4^ Internet service platforms, such as YouTube and TikTok, which have gained exponential popularity among adolescents, usually have age restrictions. Children under 13 were officially prohibited from using them independently. Uploading personal movies is considered risky because the image or movie uploaded by children can be traced by other malicious users for stalking and bullying them. Therefore, we considered uploading personal movies in addition to having met strangers in real life as risky behaviors. Response to risky behaviors was answered with “yes” or “no.”

We obtained children’s height and weight, which were measured every April by trained school nurses. Age- and sex-specific cutoff points equivalent to the adult BMI value of 25 or 18.5 kg/m^2^ for classification as overweight or thinness, respectively, were used to identify children who were overweight or thin. These cutoff points were developed by the Childhood Obesity Working Group of the International Obesity Task Force.^15^

The following questions were used to examine the children’s family and social environments: “How often do you usually interact with your parents?” and “Do you have close friends (in the real world)?” Answers to these questions were measured on a four-point scale: often (many), sometimes (several), rare (scarce), and none; then, the former two responses were collapsed. In addition, we asked, “Are there any rules at home to restrict screen time (including TV and DVD viewing, video game playing, and Internet surfing)?” The possible answers were “yes” or “no.”

### Statistical analysis

Basic characteristics, body composition, lifestyle habits, Internet time, YDQ score, Internet-related risky behaviors, and family and social factors were shown by sex. Next, Poisson regression analysis was performed to clarify the associated factors of PIU overall and stratified by sex, because our study population was relatively large and we expected that the prevalence of PIU might be high (the results from the Poisson regression model in our study were almost identical to those from the logistic regression model). Crude and adjusted prevalence ratios (PRs) and 95% confidence intervals (CIs) were calculated. In multivariate analysis, all independent variables were included with the forced entry methods. Finally, we examined the associations of Internet time with PIU and risky behaviors. All analyses were performed using STATA version 14.0, statistical software (STATA Corporation, College Station, TX, USA). A two-tailed *P* value <0.05 was considered statistically significant.

## RESULTS

In all, 13,092 returned the questionnaire (response rate 97.6%); of these, 12,130 (90.4%) children (mean age, 10.5 years; standard deviation, 0.96 years) who answered all relevant items in our analyses were included. Table 1 shows the distribution of children’s characteristics by sex. More boys got up late, skipped breakfast, used the Internet longer, and were more physically active than girls; meanwhile, girls were more likely to communicate with their parents. There was no significant difference by sex in bedtime on weekdays or in close friends. The overall prevalence of PIU was 4.2% (5.2% in boys vs 3.2% in girls); the prevalence of risky behaviors was 21.6% (31.3 vs 11.5) in spending money online, 6.6% (6.6 vs 6.6) in uploading movies, 5.2% (7.0 vs 3.1) in interpersonal issues, and 2.4% (3.5 vs 1.4) in having met strangers. More boys tended to engage in risky behaviors than girls.

**Table 1.** Characteristics of children by sex, *n* = 12,130

		Boy	Girl	Chi-square test
		% (*n* = 6,173)	% (*n* = 5,957)	*P*
Grade	4	32.6	32.0	0.561
	5	32.9	33.8	
	6	34.6	34.2	
Body composition	normal	73.1	72.7	<0.001
	overweight	17.1	14.9	
	thin	9.8	12.5	
Wakeup time on weekdays	≥7:00	5.2	3.4	<0.001
Breakfast	skipping (not every day)	9.2	8.1	0.041
Internet Time on weekdays	<2 h	61.2	75.2	<0.001
	2 to <3 h	15.2	11.2	
	3 to <4 h	9.5	5.3	
	≥4 h	14.2	8.3	
Physical activity	very often	79.3	67.0	<0.001
	often	17.3	28.5	
	rarely	3.5	4.5	
Bedtime on weekdays	<10 p.m.	66.9	65.5	0.150
	10 to 11 p.m.	26.1	27.7	
	≥11 p.m.	7.0	6.8	
Setting rule to restrict screen time	no	32.3	30.0	0.006
Interaction with parent	often or sometime	68.5	84.8	<0.001
	rare	21.6	12.0	
	none	9.9	3.2	
Close friends	scarce or none	6.3	5.7	0.125
YDQ score	0–2	83.1	89.8	<0.001
	3–4	11.7	7.1	
	≥5	5.2	3.2	
Having spent money online		31.3	11.5	<0.001
Uploading personal movies		6.6	6.6	0.995
Interpersonal issues		7.0	3.1	<0.001
Having met strangers in real life		3.5	1.4	<0.001

YDQ, Young’s diagnostic questionnaire.

Table 2 shows the associations between PIU and children’s characteristics, body composition, lifestyle, and family and social factors. In univariate analyses, boy (PR 1.63; 95% CI, 1.36–1.95), higher grades (6th, PR 1.61; 95% CI, 1.30–2.00), overweight (PR 1.61; 95% CI, 1.31–1.97), late wake up (≥7:00, PR 3.03; 95% CI, 2.46–3.73), skipping breakfast (PR 1.70; 95% CI, 1.09–2.65), longer Internet time on weekdays (2 to <3 h, PR 3.95, 95% CI, 2.98–5.23; 3 to <4 h, PR 5.62; 95% CI, 4.16–7.58; ≥4 h, PR 13.15; 95% CI, 10.52–16.45), physical activity (often, PR 1.74; 95% CI, 1.43–2.12; rarely, PR 4.90; 95% CI, 3.80–6.32), late bedtime (10 to 11 p.m., PR 1.78; 95% CI, 1.46–2.19; ≥11 p.m., PR 5.66; 95% CI, 4.55–7.03), no rules at home (PR 2.40; 95% CI, 2.02–2.85), infrequent interaction with parents (rare, PR 1.72; 95% CI, 1.39–2.13; none, PR 3.56; 95% CI, 2.82–4.49), and scarce or no close friends (PR 2.61; 95% CI, 2.04–3.35) were significantly associated with PIU. In multivariate analysis, the associations of boys (PR 1.26; 95% CI, 1.04–1.52), skipping breakfast (PR 1.43; 95% CI, 1.14–1.79), Internet time on weekdays (2 to <3 h, PR 3.49; 95% CI, 2.63–4.65; 3 to <4 h, PR 4.45; 95% CI, 3.27–6.06; ≥4 h, PR 8.25; 95% CI, 6.45–10.55), physical activity (often, PR 1.41; 95% CI, 1.15–1.73; rarely, PR 2.63; 95% CI, 2.00–3.47), late bedtime (≥11 p.m., PR 1.86; 95% CI, 1.45–2.39), no rules at home (PR 1.22; 95% CI, 1.01–1.46), no interaction with parent (PR 1.37; 95% CI, 1.06–1.77), and scarce or no close friends (PR 1.69; 95% CI, 1.30–2.19) remained significant.

**Table 2.** Poisson regression analyses on pathological Internet use, *n* = 12,130

		PIU	Univariate	Multivariate
		%	PR (95% CI)	PR (95% CI)
Sex, boy/girl		5.2/3.2	**1.63 (1.36–1.95)**	**1.26 (1.04–1.52)**
Grade	4	3.3	1	1
	5	4.0	1.23 (0.98–1.56)	0.96 (0.76–1.21)
	6	5.3	**1.61 (1.30–2.00)**	1.07 (0.85–1.34)
Body composition	normal	3.9	1	1
	overweight	6.3	**1.61 (1.31–1.97)**	0.99 (0.80–1.23)
	thin	3.2	0.82 (0.59–1.12)	0.96 (0.70–1.32)
Wakeup time on weekdays	≥7:00/<7:00	10.6/3.9	**3.03 (2.46–3.73)**	1.28 (0.96–1.70)
Breakfast	skipping/every day	10.9/3.6	**1.70 (1.09–2.65)**	**1.43 (1.14–1.79)**
Internet Time on weekdays	<2 h	1.4	1	1
	2 to <3 h	5.4	**3.95 (2.98–5.23)**	**3.49 (2.63–4.65)**
	3 to <4 h	7.6	**5.62 (4.16–7.58)**	**4.45 (3.27–6.06)**
	≥4 h	17.8	**13.15 (10.52–16.45)**	**8.25 (6.45–10.55)**
Physical activity	very often	3.2	1	1
	often	5.5	**1.74 (1.43–2.12)**	**1.41 (1.15–1.73)**
	rarely	15.6	**4.90 (3.80–6.32)**	**2.63 (2.00–3.47)**
Bedtime on weekdays	<10 p.m.	2.8	1	1
	10 to 11 p.m.	4.9	**1.78 (1.46–2.19)**	1.15 (0.93–1.41)
	≥11 p.m.	15.6	**5.66 (4.55–7.03)**	**1.86 (1.45–2.39)**
Setting rule to restrict screen time	no/yes	7.0/2.9	**2.40 (2.02–2.85)**	**1.22 (1.01–1.46)**
Interaction with parents,	often or sometime	3.3	1	1
	rare	5.6	**1.72 (1.39–2.13)**	1.06 (0.85–1.32)
	none	11.6	**3.56 (2.82–4.49)**	**1.37 (1.06–1.77)**
Close friends	scarce or none	10.0/3.8	**2.61 (2.04–3.35)**	**1.69 (1.30–2.19)**

CI, confidence interval; PIU, pathological Internet use; PR, prevalence ratio.

In multivariate analysis, all variables were simultaneously entered.

In Table 3, the results from multivariate Poisson regression analyses of PIU were shown by sex. Beside Internet time for both sexes, skipping breakfast (PR 1.36; 95% CI, 1.02–1.83), physical activity (often, PR 1.62; 95% CI, 1.25–2.10; rarely, PR 3.32; 95% CI, 2.32–4.74), late bedtime (≥11 p.m., PR 1.77; 95% CI, 1.29–2.42), no rules at home (PR 1.27; 95% CI, 1.01–1.60), and scarce or no close friends (PR 1.44; 95% CI, 1.03–2.01) were significantly associated with PIU in boys, while late wake up (≥7:00, PR 1.63; 95% CI, 1.02–2.60), skipping breakfast (PR 1.46; 95% CI, 1.02–2.10), physical activity (rarely, PR 1.75; 95% CI, 1.11–2.74), late bedtime (≥11 p.m., PR 2.04; 95% CI, 1.35–3.08), and scarce or no close friends (PR 2.24; 95% CI, 1.47–3.43) were significantly associated. Comparing the differences between boys and girls, higher PRs were seen with infrequent physical activity in boys and with no close friends in girls.

**Table 3.** Poisson regression analyses on pathological Internet use by sex, *n* = 12,130

		Boys		Girls	

		PIU	Multivariate	PIU	Multivariate
		%	PR (95% CI)	%	PR (95% CI)
Grade	4	4.6	1	1.9	1
	5	4.9	0.87 (0.65–1.16)	3.1	1.16 (0.77–1.77)
	6	6.0	0.98 (0.74–1.28)	4.5	1.25 (0.84–1.88)
Body composition	normal	4.7	1	3.1	1
	overweight	7.6	1.01 (0.77–1.33)	4.7	0.93 (0.66–1.32)
	thin	4.8	1.14 (0.77–1.69)	1.9	0.73 (0.42–1.27)
Wakeup time on weekdays	≥7:00/<7:00	10.5/4.9	1.12 (0.78–1.63)	10.8/2.9	**1.63 (1.02–2.60)**
Breakfast	skipping/every day	11.0/4.6	**1.36 (1.02–1.83)**	10.7/2.5	**1.46 (1.02–2.10)**
Internet Time on weekdays	<2 h	1.8	1	1.0	1
	2 to <3 h	5.9	**3.05 (2.12–4.37)**	4.6	**4.12 (2.59–6.55)**
	3 to <4 h	7.7	**3.70 (2.52–5.45)**	7.6	**5.93 (3.57–9.86)**
	≥4 h	17.6	**6.80 (4.99–9.28)**	18.3	**10.93 (7.33–16.29)**
Physical activity	very often	3.9	1	2.4	1
	often	8.2	**1.62 (1.25–2.10)**	3.9	1.11 (0.80–1.54)
	rarely	21.1	**3.32 (2.32–4.74)**	11.2	**1.75 (1.11–2.74)**
Bedtime on weekdays	<10 p.m.	3.6	1	1.9	1
	10 to 11 p.m.	6.2	1.16 (0.89–1.51)	3.6	1.14 (0.80–1.62)
	≥11 p.m.	17.2	**1.77 (1.29–2.42)**	13.8	**2.04 (1.35–3.08)**
Setting rule to restrict screen time	no/yes	8.5/3.6	**1.27 (1.01–1.60)**	5.4/2.3	1.15 (0.85–1.57)
Interaction with parents,	often or sometime	4.0	1	2.6	1
	rare	6.1	1.05 (0.80–1.37)	4.6	1.05 (0.71–1.55)
	none	11.3	1.32 (0.97–1.79)	12.5	1.58 (0.97–2.56)
Close friends	scarce or none	11.8/4.8	**1.44 (1.03–2.01)**	8.0/2.9	**2.24 (1.47–3.43)**

CI, confidence interval; PIU, pathological internet use; PR, prevalence ratio.

In multivariate analysis, all variables were simultaneously entered.

**Figure 1.**
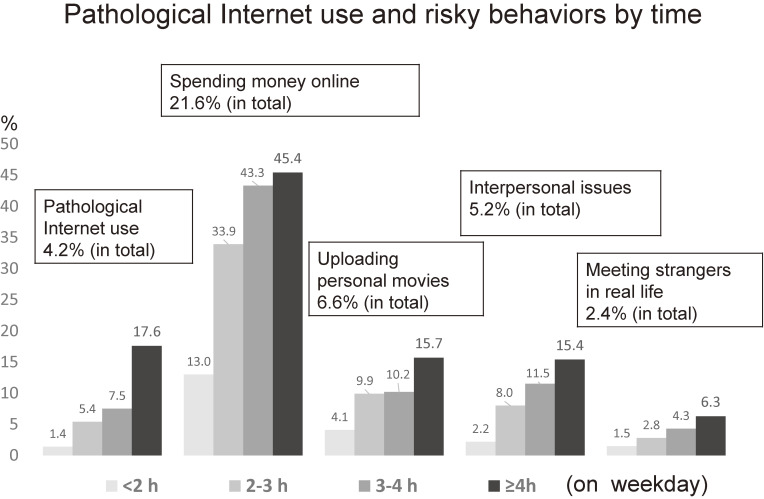
Prevalence of pathological Internet use (PIU) and risky behaviors by time. The associations between PIU, Internet-risky behaviors, and Internet time show a different trend. In the rate of PIU, there was a big difference between Internet time 3 to <4 hours (7.5%) and ≥4 hours (17.6%), while big differences existed between <2 hours and ≥2 hours in the rates of spending money online (13.0% vs 33.9%), uploading personal movies (4.1% vs 9.9%), and interpersonal issues (2.2% vs 8.0%).

Figure 1 shows the prevalence of PIU and risky behaviors by time. Children with Internet time <2 hours had the lowest rates, while those with ≥4 hours had the highest rates in PIU and all risky behaviors. However, an increase in trends differed. In the rate of PIU, there was a big difference between Internet time ≥4 hours (17.6%) and 3 to <4 hours (7.5%), while big differences existed between <2 hours and ≥2 hours in the rates of spending money online, uploading personal movies, and interpersonal issues.

**Figure 2.**
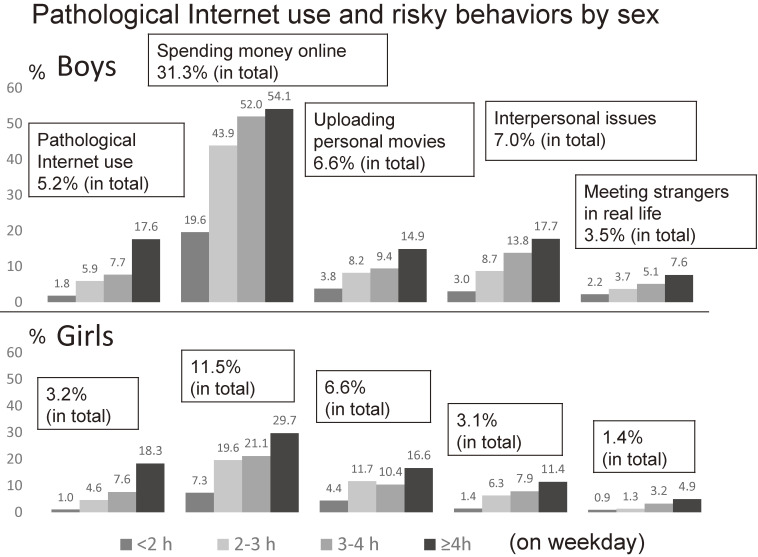
Prevalence of pathological Internet use and risky behaviors by sex. Similar trends were seen between boys and girls. However, spending money online was much prevalent in boys, especially for those using the Internet for more than 2 hours. Notably, 1.4% of girls had met strangers encountered online in real life.

Figure 2 shows the prevalence of PIU and risky behaviors by sex. Similar trends were seen between boys and girls. However, spending money online was much more prevalent in boys, especially for those using the Internet for ≥2 hours.

## DISCUSSION

We conducted the large-scale school-based study on Internet use in the elementary school, which is rare while the number of studies in college and in high and junior high school have been increasing. Our study showed that PIU and risky behaviors were not rare among elementary school children, and that PIU was associated with their own unhealthy lifestyles including prolonged Internet use, late bedtime, physical inactivity, skipping breakfast, as well as family and social environments such as no rules at home, no close friends in real life, and no child-parent interaction. To the best of our knowledge, this is the first study clarifying the association comprehensively between PIU, lifestyles, and family and social environments among elementary school children. For parents or guardians, setting rules at home, having enough interaction with children, and helping them to develop friendships with other children in real life were considered preventive measures from PIU.

The prevalence rate of PIU in elementary school children was 4.2% in our study, which was in line with other studies in Asia, such as 6.3% in China and 3.6% in Japan,^7^^,^^11^ in which a survey was also conducted with YDQ.^2^ Although it is difficult to compare the prevalence of PIU worldwide due to different social and cultural contexts, and the prevalence rates in Eastern Asian countries were known to be higher than those in Western countries.^5^ Asian countries need to address children’s Internet-related problems more seriously.

There have been no consistent results of sex differences in adolescent PIU. Although many studies showed male dominance in PIU,^5^^,^^6^^,^^16^ some studies demonstrated female dominance.^8^^,^^14^ In our study targeting elementary school children, more boys were likely to contract PIU than girls (5.2% vs 3.2%). This may stem from the fact that boys were more likely to use online games while girls, especially in junior school, were more likely to be absorbed in social networking and texting, such as Line and Twitter.^8^^,^^14^^,^^17^ Takahashi et al showed boys’ dominance in elementary school and girls’ dominance in junior high school on PIU.^7^ According to the national survey of elementary school children in Japan in 2018,^4^ smartphone ownership was only 35.9% and game console ownership was 65.3%. The high rate of game ownership in elementary school could cause boys’ dominance of PIU in our study.

We assessed the association of lifestyle (sleep, breakfast, physical activity, and Internet time) with PIU and demonstrated that PIU had a significant association with skipping breakfast, physical inactivity, and late bed time, even though prolonged Internet time, which had the strongest association, was simultaneously inserted in multivariate analysis. This result showed the importance of regular lifestyles as early detection or countermeasure to PIU. Our findings were consistent with previous studies on sleep and dietary disturbance and physical inactivity.^10^^,^^18^^,^^19^ In addition to restriction on Internet time, fundamental lifestyles of children should be heeded.

Our study also demonstrated the importance of family factors: no rules at home and infrequent child-parent interaction were significantly associated with PIU in a multivariate model. There have been many previous studies showing significant association between screen time of children and setting rule at home,^20^^–^^22^ and one study exploring the association with PIU.^23^ Bonnaire et al investigated the association in adolescents (mean age, 13.2 years) and stated that rules about gaming use could prevent PIU in males and restrictions could prevent PIU in females. Our findings from the large-scale study can support the effectiveness of setting rules to prevent elementary school children from PIU. Regarding child-parent interaction, our results were consistent with other reports.^24^^–^^26^ Schneider et al stated that maternal and paternal relationships were negatively associated with problem gaming. The importance of parents was also shown in intervention or treatment for PIU.^27^^,^^28^ Han et al stated that family cohesion may be an important factor in the treatment of PIU from the intervention study assessing patterns of brain activation in response to parental love. Parental involvement needs to be considered as a critical countermeasure in addressing children’s PIU.

As a social factor, scarce or no close friends in real life was associated with PIU in our study. Previous studies on friends of children with PIU were limited.^6^^,^^8^^,^^29^ Kojima et al demonstrated that junior high school students lacking person of trust to talk to were more likely to be PIU, and Stavropoulos et al indicated that students spending more daily time online than with their closest relationships in the classroom were associated with PIU.^30^ Our results support the importance of peers in line with previous literature.

Moreover, we conducted stratified analyses by sex and found that infrequent physical activity in boys and no close friends in girls showed stronger associations with PIU than the opposite sex (in Table 3). We did not infer that having close friends was less important to prevent PIU in boys than in girls. Boys were reported to make many friends when they were physically active,^31^ and to have a stronger correlation of physical activity level among same-sex friends than girls.^32^ Thus, we assumed that in our study, the PR of no close friends in boys would be lowered owing to the confounding effect of physical activity. We believe that having close friends in real life is equally important to boys and girls to prevent PIU. This study was the first to analyze associated factors of PIU by sex; it also demonstrated the importance of friendship in PIU prevention among elementary school children. It should be recommended for parents or guardians to establish an environment where children can develop real-life friendships with peers.

We also found that online risky behaviors, especially spending money online, were common in elementary school children. Notably, the prevalence of having met strangers encountered online in real life was 2.4% (3.5% in boys vs 1.4% in girls) in our study. The national report also demonstrated that the prevalence was 0.9% in elementary school children and 4.3% in junior high school students.^4^ Parents and guardians must consider meeting strangers in real life as becoming common behavior in elementary school children. In the relationship between the prevalence of PIU and risky behaviors and Internet time (Figure 1), the different trends were seen. Children who spent ≥4 hours a day on the Internet had much higher rates of PIU than those with fewer hours. Given that PIU is also known as “Internet addiction,” this trend seems reasonable. Meanwhile, in other risky behaviors, such as spending money online, uploading personal movies, and causing interpersonal issues, children who spent 2 to 3 hours a day on the Internet had much higher rates of these risky behaviors than children with fewer hours. Given this difference, we expect that preventive education concerning online risky behaviors might need a different approach from PIU; instead of limiting time of Internet use, education for safety use should be implemented for all children.

The current study has several limitations. First, our data on PIU and risky behaviors stemmed from self-reported questionnaires in elementary schools. Therefore, they might be susceptible to information bias, such as social desirability bias. Children might report shorter Internet time, earlier bedtime, and lower score of PIU than they actually did. Thus, our study may have underestimated PIU prevalence and the association between PIU and unhealthy lifestyles. Second, our study was a cross-sectional design, which does not allow for causality. However, paying attention to the daily lifestyles and family and social environments of children is, in any case, useful for early detection of health problems. Third, we conducted a large-scale study (61.1% of elementary schools in Toyama were involved) with extremely high response rates (97.6%); however, all participants were from one prefecture in Japan. Caution is needed in generalizing our findings beyond that prefecture. In the future, a nationwide representative or longitudinal study among elementary school children should be conducted.

### Conclusions

Our results showed that PIU and online related risky behaviors were not rare in elementary school children. In addition to unhealthy individual lifestyles, family and social factors—infrequent child-parent interaction and having no close friends in real life—were associated with PIU. While the rate of PIU was much higher in prolonged Internet use, risky behaviors were seen prevalent among non-absorbed Internet users. For parents or guardians, increasing child-parent interaction and helping children develop close friendships in real life are effective deterrents to PIU.
